# Draft Genome Sequence of a Clinical Isolate of Fusarium fujikuroi Isolated from a Male Patient with Acute Myeloid Leukemia

**DOI:** 10.1128/genomeA.00476-18

**Published:** 2018-06-21

**Authors:** Camilla Urbaniak, Sanjeet Dadwal, Karine Bagramyan, Kasthuri Venkateswaran

**Affiliations:** a Jet Propulsion Laboratory, California Institute of Technology, Pasadena, California, USA; b City of Hope National Medical Center, Duarte, California, USA; c Beckman Research Institute, City of Hope National Medical Center, Duarte, California, USA

## Abstract

Here, we present the draft whole-genome sequence of a clinical isolate of Fusarium fujikuroi cultured from a patient undergoing chemotherapy for refractory acute myeloid leukemia.

## GENOME ANNOUNCEMENT

Fusarium was first discovered in 1958, and while notorious for causing the devastating disease it causes in plants and crops, it is now recognized as an emerging global opportunistic human pathogen causing numerous cases of fusariosis every year (1, 2). While roughly 80% of the human cases of fusariosis are caused by members of the Fusarium oxysporum and Fusarium solani species complexes, there are many infections attributed to the Fusarium fujikuroi species complex. Here, we report the draft whole-genome sequence of F. fujikuroi strain COH1152, which was isolated from a 68-year-old male with refractory acute myeloid leukemia. While undergoing chemotherapy, he developed a large lesion on his left leg after sustaining minor trauma, followed by neutropenic fever and multiple small painful nodules on his face, abdomen, and thighs. The biopsy sample of the left thigh skin lesion showed invasive hyphal elements, and a Fusarium sp., as identified by morphology, was isolated from both blood and tissue cultures. A Fusarium isolate, cultured from the blood and denoted COH1152, was sent for whole-genome sequencing and identified as the species F. fujikuroi.

The COH1152 genome was paired-end sequenced (2 × 100 bp) on the Illumina HiSeq platform with a 350-bp insert size, resulting in a total of 41 million reads (GC content, 47.5%). Trimmomatic, on the Galaxy server (https://usegalaxy.org), was used to remove the sequencing adaptors (settings, max mismatch = 2, how accurate the match between the two “adaptor ligated” reads = 30, and how accurate the match between any adaptor = 10) and to trim the leading and trailing ends (setting, minimum quality required to keep a base = 3). Postprocessed reads were *de novo* assembled with ABySS version 2.0.2 (3), using a k-mer size of 88. The COH1152 genome assembly resulted in a genome size of 48 Mb, with an *N*_50_ value of 1,212,708 bp. The number of scaffolds generated was 4,495, with a max scaffold length of 6,034,165 bp. The number of scaffolds over 1 kb was 501.

A second clinical Fusarium isolate, COH1141, as identified by morphology, was cultured from another patient also receiving chemotherapy for acute myeloid leukemia. However, the isolate that was sent for whole-genome sequencing was probably a laboratory contaminant of Aspergillus niger. We, however, have chosen to upload its draft genome sequence for public accessibility (k-mer size, 89; genome size, 32 Mb; number of scaffolds over 1 kb, 350).

### Accession number(s).

The assembled whole-genome sequences have been deposited in DDBL/EMBL/GenBank under the accession numbers QBDQ00000000 (Fusarium fujikuroi COH1152) and QBDR00000000 (Aspergillus niger COH1141). The sequences have also been deposited in the NASA GeneLab and can be found online (https://genelab-data.ndc.nasa.gov/genelab/accession/GLDS-177/). This is the first version.

## References

[1] Gordon TR 2017 *Fusarium oxysporum* and the *fusarium* wilt syndrome. Annu Rev Phytopathol 55:23–39. doi:10.1146/annurev-phyto-080615-095919. 28489498

[2] Al-Hatmi AMS, Hagen F, Menken SBJ, Meis JF, de Hoog GS 2016 Global molecular epidemiology and genetic diversity of *Fusarium*, a significant emerging group of human opportunists from 1958 to 2015. Emerg Microbes Infect 5:e124. doi:10.1038/emi.2016.126. 27924809 PMC5180370

[3] Simpson JT, Wong K, Jackman SD, Schein JE, Jones SJM, Birol İ 2009 ABySS: a parallel assembler for short read sequence data. Genome Res 19:1117–1123. doi:10.1101/gr.089532.108. 19251739 PMC2694472

